# Supplementary material for: A fault-intrusion-tolerant system and deadline-aware algorithm for scheduling scientific workflow in the cloud
Source: PeerJ Comput Sci. 2021 Nov 2;7:e747. doi: 10.7717/peerj-cs.747 (PMC8576554; doi:10.7717/peerj-cs.747)
Supplement: Supplemental Information 6 [file peerj-cs-07-747-s006.zip › Myproject1.1/docs/org/cloudbus/cloudsim/network/datacenter/AggregateSwitch.html]

AggregateSwitch


JavaScript is disabled on your browser.


- Overview
- Package
- Class
- Use
- Tree
- Deprecated
- Index
- Help

- Prev Class
- Next Class

- Frames
- No Frames

- All Classes

- Summary:
- Nested |
- Field |
- Constr |
- Method

- Detail:
- Field |
- Constr |
- Method


org.cloudbus.cloudsim.network.datacenter

## Class AggregateSwitch

- java.lang.Object
- - org.cloudbus.cloudsim.core.SimEntity
  - - org.cloudbus.cloudsim.network.datacenter.Switch
    - - org.cloudbus.cloudsim.network.datacenter.AggregateSwitch

- All Implemented Interfaces:
  :   java.lang.Cloneable

  ---

    

  ```
  public class AggregateSwitch
  extends Switch
  ```

  This class allows to simulate aggregate switch for Datacenter network. It interacts with other
  switches in order to exchange packets.
  Please refer to following publication for more details:
  Saurabh Kumar Garg and Rajkumar Buyya, NetworkCloudSim: Modelling Parallel Applications in Cloud
  Simulations, Proceedings of the 4th IEEE/ACM International Conference on Utility and Cloud
  Computing (UCC 2011, IEEE CS Press, USA), Melbourne, Australia, December 5-7, 2011.

  Since:
  :   CloudSim Toolkit 1.0

- - ### Field Summary

    - ### Fields inherited from class org.cloudbus.cloudsim.network.datacenter.Switch

      `BagofTaskVm, datacenterid, dc, downlinkbandwidth, downlinkswitches, downlinkswitchpktlist, fintimelistHost, fintimelistVM, hostlist, id, latency, level, numport, packetTohost, pktlist, switching_delay, uplinkbandwidth, uplinkswitches, uplinkswitchpktlist, Vmlist`
    - ### Fields inherited from class org.cloudbus.cloudsim.core.SimEntity

      `FINISHED, HOLDING, RUNNABLE, WAITING`
  - ### Constructor Summary

    Constructors

    Constructor and Description

    `AggregateSwitch(java.lang.String name,
    int level,
    NetworkDatacenter dc)`

    Constructor for Aggregate Switch We have to specify switches that are connected to its
    downlink and uplink ports, and corresponding bandwidths
  - ### Method Summary

    Methods

    | Modifier and Type | Method and Description |
    | --- | --- |
    | `protected void` | `processpacket_down(SimEvent ev)` Send Packet to switch connected through a downlink port |
    | `protected void` | `processpacket_up(SimEvent ev)` Send Packet to switch connected through a uplink port |

    - ### Methods inherited from class org.cloudbus.cloudsim.network.datacenter.Switch

      `getfreehostlist, getfreeVmlist, getHostwithVM, processEvent, processhostpacket, processpacket, processpacketforward, shutdownEntity, startEntity`
    - ### Methods inherited from class org.cloudbus.cloudsim.core.SimEntity

      `cancelEvent, clone, getEventBuffer, getId, getName, getNextEvent, getNextEvent, getState, numEventsWaiting, numEventsWaiting, pause, run, schedule, schedule, schedule, schedule, scheduleFirst, scheduleFirst, scheduleFirst, scheduleFirst, scheduleFirstNow, scheduleFirstNow, scheduleFirstNow, scheduleFirstNow, scheduleNow, scheduleNow, scheduleNow, scheduleNow, selectEvent, send, send, send, send, sendNow, sendNow, sendNow, sendNow, setEventBuffer, setId, setState, waitForEvent`
    - ### Methods inherited from class java.lang.Object

      `equals, finalize, getClass, hashCode, notify, notifyAll, toString, wait, wait, wait`

- - ### Constructor Detail


    - #### AggregateSwitch

      ```
      public AggregateSwitch(java.lang.String name,
                     int level,
                     NetworkDatacenter dc)
      ```

      Constructor for Aggregate Switch We have to specify switches that are connected to its
      downlink and uplink ports, and corresponding bandwidths

      Parameters:
      :   `name` - Name of the switch
      :   `level` - At which level switch is with respect to hosts.
      :   `dc` - Pointer to Datacenter
  - ### Method Detail


    - #### processpacket\_down

      ```
      protected void processpacket_down(SimEvent ev)
      ```

      Send Packet to switch connected through a downlink port

      **Overrides:**
      :   `processpacket_down` in class `Switch`

      Parameters:
      :   `ev` - Event/packet to process


    - #### processpacket\_up

      ```
      protected void processpacket_up(SimEvent ev)
      ```

      Send Packet to switch connected through a uplink port

      **Overrides:**
      :   `processpacket_up` in class `Switch`

      Parameters:
      :   `ev` - Event/packet to process


- Overview
- Package
- Class
- Use
- Tree
- Deprecated
- Index
- Help

- Prev Class
- Next Class

- Frames
- No Frames

- All Classes

- Summary:
- Nested |
- Field |
- Constr |
- Method

- Detail:
- Field |
- Constr |
- Method
